# ‘Eating is like experiencing a gamble’: A qualitative study exploring the dietary decision‐making process in adults with inflammatory bowel disease

**DOI:** 10.1111/hex.13873

**Published:** 2023-09-20

**Authors:** Yin Ting‐Ting, Tu Wen‐Jing, Li Yi‐Ting, Xu Wen‐Jing, Xu Gui‐Hua

**Affiliations:** ^1^ College of Nursing Nanjing University of Chinese Medicine Nanjing China

**Keywords:** decision‐making, dietary, inflammatory bowel disease, qualitative research

## Abstract

**Background:**

For adults with inflammatory bowel disease (IBD), they experience many challenges in dietary decision‐making. Thus, this study examined the perspective and experiences of adults with IBD in dietary decision‐making.

**Objective:**

This study aimed to explore the perception and consideration of people with IBD in their daily dietary decisions through monitoring, interpretation and action during the decision‐making process.

**Design:**

A qualitative study of individuals affected by IBD was conducted through semistructured interviews.

**Results:**

Twenty patients were recruited from four tertiary hospitals in Nanjing, China, and each participant completed a semistructured interview. The majority of participants reported on the process and experience of dietary decision‐making. Key themes were categorised into three stages: (1) assessing needs, preferences and food cues (monitor); (2) moving from experience to expertise (interpret) and (3) balancing expectations amidst limitations (act). The majority of participants reported that their decisions were shaped by assessing current disease status and food cues. Those interviewed with IBD were willing to make tradeoffs for bowel stability, but their decisions were also influenced by past dietary experiences and traditional Chinese beliefs. The lack of awareness of dietary guidelines was a significant barrier to healthy eating decisions. Positive or negative feelings accompanied dietary decisions.

**Conclusion:**

Although most people with IBD change their diet after diagnosis, the changes made are often inconsistent with existing dietary recommendations. Several factors can influence the dietary decision‐making process. This study will help assess the experiences of people with IBD in dietary decision‐making to encourage the formation of targeted dietary health and well‐being interventions. Knowledge of nutrition and diet should be provided in education and training programmes for IBD management.

**Patient or Public Contribution:**

The first three authors of this paper were the lead researchers in this study's design. These authors were mentored by patient researchers who also contributed to the manuscript, and the research process was co‐lead and directed by other patient participants and consultants. The results of this paper were directly obtained from patient participants.

## 1 INTRODUCTION

Inflammatory bowel disease (IBD) primarily includes ulcerative colitis (UC) and Crohn's disease (CD). IBD is a lifelong disease and chronic inflammation of the gastrointestinal tract, which can lead to diarrhoea, abdominal pain, rectal bleeding, weight loss and nutritional deficiencies. In China, the incidence of IBD has continued to increase in recent years.^1^ Growing evidence shows that diet plays an important role in the prognosis of IBD. Individualised dietary formulation, abolishing misconceptions in dietary beliefs and practices and ensuring adherence to an anti‐inflammatory diet could be quintessential in the achievement of deep remission in IBD. Thus, diet management is central to the self‐management of patients with IBD.^2^ The Asian Diet and Inflammatory Bowel Disease Working Group guidelines suggest that once the disease is in remission, there is no need for dietary modification or restriction, and that the patient can continue to eat as normal as any other family member.^3^ Although scientific evidence convincingly links dietary content to health, dietary decision‐making is a complex process that involves more than just considering the nutritional value of food. Dietary decision‐making also varies by disease as well as by cultural and contextual factors. In addition, nutrition is a specialised science. Patients with IBD as nonprofessionals tend to make high‐frequency daily dietary decisions, which may lead to unscientific dietary behaviour. Diet restriction, diet avoidance and diet exclusion are frequently reported in patients with IBD.^4^, ^5^, ^6^ Notably, a vast gap could be observed between the theoretically proposed dietary specifications in IBD and their implementation by patients in day‐to‐day practice behaviour.

**Figure 1:**
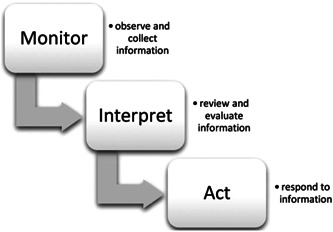
Conceptual model of the three processes involved in everyday decision‐making: monitoring, interpreting and acting.^9^

Behaviour and decision‐making are closely linked to each other.^7^ Thus, the role of dietary decision‐making cannot be undermined in attaining healthy dietary behaviour. Nevertheless, little is known about how adults with IBD make dietary decisions. Naturalistic decision‐making is how decisions are made in dynamic, ill‐defined and uncertain situations that involve shifting goals and high stakes, and this process may involve other actors.^8^ Figure 1 employs a typical naturalistic decision‐making process.^9^ Notably, the eating behaviour of patients with IBD is also a complex decision‐making process in the face of certain or uncertain situations. Patients make diet decisions everyday, such as meal times, ingredient preparation and dining venues, which have immediate and long‐term consequences that affect their symptom burden, disease prognosis and significant events such as unplanned hospitalisations. Considering that dietary decisions in people with IBD fit into the realm of naturalistic decision‐making, a conceptual model of the three decision‐making processes of the naturalistic decision theory can be used to provide a comprehensive understanding of ‘how a decision is made’ and ‘why it is made’ by a person with IBD in a real‐life dietary decision‐making situation.

Based on the above‐mentioned background, this study adopts a qualitative research approach to explore the process and experience of daily dietary decision‐making from patients' perspectives and to understand the pathways of patients' judgement and food choices. An attempt was made to provide new perspectives and strategies for designing effective intervention programmes for healthy eating behaviours and to address personal health problems caused by poor dietary decisions in people with IBD.

## 2 MATERIALS AND METHODS

### 2.1 Study design and ethics

This study aimed to understand the experiences of people with IBD throughout the dietary decision‐making process. All subjects provided informed consent before participation in the study. The study was conducted in accordance with the Declaration of Helsinki, approved by the Ethics Committee of the affiliated hospital, and completed registration in the Chinese Clinical Trials Registry.

### 2.2 Participants and recruitment

Participants were initially selected using purposive sampling based on pre‐established eligibility criteria. Sampling continued until the interview subject was saturated and then stopped. The participants were derived from consecutive outpatients with a confirmed diagnosis of CD or UC who were treated or followed up in IBD wards of four tertiary general hospitals in Nanjing, China. Participants aged at least 18 years met the inclusion criteria. Pregnant and lactating women were excluded from the study. Patients were recruited between April 2022 and May 2023. Data were collected and summarised in Microsoft Excel. Demographic information, including age, gender, marital status, education, occupation, place of residence, weekly frequency of eating out (times/week), past and current surgical interventions, medications, type of IBD and duration of the disease, medicaments and disease activity at the time of examination were recorded. Nutritional status was recorded with regard to current weight, height, body mass index, the presence of food allergies or intolerances and the use of dietary supplements. Disease activity was assessed using the clinical disease activity index for CD and the Mayo scale for UC after an accurate clinical assessment by an experienced IBD clinician.

### 2.3 Interview procedure

Interested individuals were invited to send a message to the principal investigator via WeChat. WeChat is currently regarded as the widely used social media platform for the general public in China. WeChat supports not only messaging and free calling but also all kinds of voice, photos and video calls. The researcher confirmed the eligibility of participants and scheduled interviews. We conducted five face‐to‐face field interviews. Fifteen interviews were conducted online via WeChat or over the phone because of the COVID‐19 pandemic. The use of WeChat to recruit patients is a single line of peer‐to‐peer contact with the patient, with no impact on the patient's personal information security or ethical aspects. Interviews lasted between 39 and 152 min, with an average of 70 min. We used Chinese for the interviews. The original Chinese version of the interview was translated by the authors. Then, a fluently bilingual (English and Chinese) professor performed a backtranslation. Both versions were compared, and discrepancies were analysed to clarify the Chinese interview. All informed consents were obtained using a paper and pencil survey before the interview. The current study is part of an ongoing project, and several papers have been written based on the same sample. Interviews followed a semistructured format that was developed on the basis of research objectives, previous research and clinical observations. The interview guide included open‐ended and survey‐style questions (see details in Supporting Information: Appendix). The reliability of the interview guide was determined by conducting three pretests before the main study, and three of the questions were slightly modified as suggested. Considering that no major changes were made to the interview outline, the revised outline had no effect on the overall thrust; therefore, the three preinterviews were included in the final analysis.

### 2.4 Data analysis

Interview recordings were transcribed verbatim using automated software. Transcripts were edited manually for accuracy. After transcription, the data were analysed in accordance with the principle of Colaizzi seven‐step analysis. The research team followed Colaizzi seven‐step analysis for data analysis, which includes the following processes: (1) repeatedly listening to the audio recordings and carefully reading the interview texts; (2) summarising and collecting recurring ideas; (3) coding the ideas repeatedly mentioned by the interviewees; (4) collecting and coding these ideas and creating a coding manual; (5) describing in detail what the interviewees described; (6) identifying similar ideas and then summarising and sublimating them into new themes and (7) reviewing the content with the interviewees to validate and capture additional information. Interviews were summarised in Microsoft Word to facilitate data storage and encoding. In this study, data collection and analysis were carried out concurrently to explore emerging themes from the remaining interviews. Data analysis was conducted in collaboration with two authors, and validated by another author, who has experience in qualitative research. The researcher initially read through the interview transcripts, repeatedly, to obtain a general understanding. The researcher then coded the interviews using an extensive coding scheme (open coding). The codes for each interview were revised, reviewed and categorised into categories and subcategories. Emerging categories and subcategories were edited to avoid overlap amongst categories and excessive heterogeneity within individual categories When no new information came to light, the research team stopped, including study participants. Ultimately, 20 study participants participated in the interviews, which is consistent with the principle of data saturation.

### 2.5 Rigour

The COREQ checklist was followed throughout the study.^10^ The quality and credibility of the results were ensured by adopting various methods. First, researchers began establishing contact with participants in November 2021 to gain patient trust and build rapport. Second, all interviews were conducted by one lead author. The interview was conducted by the first author, a researcher who systematically studied the relevant theories of qualitative research and repeatedly practiced and confirmed that she has mastered the skills of qualitative interviews. Finally, throughout data analysis, two researchers independently completed transcription to ensure that no personal biases influence translation. The two authors involved in the analysis met regularly to discuss the synthesis of data, refining themes and reflecting on how their individual professional backgrounds and experiences influenced the interpretation and portrayal of the data.

## 3 RESULTS

A total of 22 respondents were approached and consented to be included in the present study. Data saturation was achieved after 20 interviews with recruitment subsequently closing. Most interview participants worked in metropolitan areas (*n* = 13). Among the 20 participants, 13 had UC, and 7 had CD. Other participant characteristics are shown in Table 1.

**Table 1:** Patient participant characteristics (*N* = 20).

Characteristics	*n*	(%)	Mean	SD
Age			34.20	7.50
Gender				
Male	11	55%		
Female	9	45%		
Marital status				
Single	7	35%		
Married	13	65%		
Body mass index (kg/m^2^)			21.05	2.51
Education				
Illiteracy	1	5%		
Primary school	1	5%		
High school or technical school	7	7 (35%)		
University	11	11 (55%)		
Occupation				
Employee	15	75%		
Students	2	10%		
Unemployed	3	15%		
Region				
Urban	13	65%		
Rural	7	35%		
Food allergy or intolerance				
Yes	5	25%		
No	9	45%		
Nonreported	6	30%		
Weekly frequency of eating out (times/week)				
1–3	3	15%		
4–6	12	60%		
≥7	5	25%		
Duration of disease (years)			6.25	6.50
Type of inflammatory bowel disease				
Ulcerative colitis	13	65%		
Crohn's disease	7	35%		
Disease activity				
Active	11	55%		
Remission	9	45%		
Surgery				
Yes	6	30%		
No	14	70%		
Medication				
Yes	19	95%		
No	1	5%		
Additional nutritional product				
Yes	9	45%		
No	11	55%		

In this qualitative study, deep experiences and feelings of people with IBD were derived on the basis of daily dietary decision‐making using Colaizzi seven‐step analysis (Table 2).

**Table 2:** Major themes and minor themes.

Major themes	Minor themes
(1) Monitoring phase of decision‐making	
Assessing needs, preferences and cues	Gastrointestinal symptoms and nutritional status
	Food clues
(2) Interpreting phase of decision‐making	
Moving from experience to expertise	Dietary guidelines
	Self‐experiences
	Traditional Chinese food cultures
(3) Acting phase of decision‐making	
Balancing expectations amidst limitations	Positive adjustment: repeated trials to find tolerable foods
	Self‐limitation: Limit or avoid some foods
	Static diets: no changes postdiagnosis
(4) Psychological experience of the dietary decision‐making process	
Positive perception	Keeping hope and enjoying
Negative perception	Uncertainty

## 4 THEME

### 4.1 ‘Healthy food is a priority for me to keep my gut stable’: Assessing needs, preferences and cues (Monitor)

#### 4.1.1 Gastrointestinal symptoms and nutritional status

In patients with IBD, gastrointestinal symptoms such as abdominal pain and diarrhoea are a barometer of patient's dietary decisions. Participants described monitoring with regard to tracking health information physiologically, including gastrointestinal symptoms and nutritional status. Patients mentioned that weight and intestinal symptoms were closely monitored and that these changes caused patients to select certain foods as supplements.

I have a narrow intestinal tract and I choose my vegetables carefully. (man, 37 years)

I believe that, when you are craving something, it means your body is lacking it, and you should supplement it as soon as possible. My legs cramp up at night when I sleep, and I realize I need to take more calcium tablets for calcium. (female, 44 years)

#### 4.1.2 Food clues

Respondents also reported a generally high sensitivity to visual food cues in menu choices. The suitability of the cooking style and nutritional content of the food is considered during the monitoring phase of dietary decision‐making.

Canteen or takeaway dishes are very suitable for young people's taste, fried or spicy, for me, I think these are unhealthy foods. (male, 60 years)

Before I got sick, I ate whatever I wanted, whatever tasted good. And now. I don't have a strong desire for tasty food anymore. As long as it is good for my body and makes me feel comfortable, I will keep eating it. I choose something steamed or boiled, without seasoning or spices. (male, 22 years)

I would prefer to have meat and vegetables, with a reasonable food mix, and protein is a must, and I drink a lot of soup on our side and I think it's good for the intestines. (female, 19 years)

Some patients also indicated that price was an important factor that must be considered during food selection.

I have to think about the price of food, you know, having this disease has already made it difficult for me to make ends meet. (female, 52 years)

### 4.2 ‘I'm an expert in IBD dietary management’: Moving from experience to expertise (Interpret)

#### 4.2.1 Dietary guidelines

Based on the patient's description, the surgeon and dietitian gave dietary advice to the patient during the episode based on evidence‐based dietary guidelines.

The reason for taking nutritional powders is that my doctor recommended that I take them, and I think the doctor's advice is more scientific.I realised that diet plays an important role in my body, and I started to make sure that my diet is suitable for gut. (female, 23 years)

The dietitian also recommended increasing protein intake, and I increased my fish intake and focused on protein when I ate. (female, 36 years)

Patients demonstrate that they rely on information from online resources (e.g. nutrition websites and social media) or unsolicited advice from health care providers.

Decide what you eat by reading specialized books, literature about diet, or websites. (male, 41 years)

There's a Mint Health app where you can look up nutrients in food, and that's very useful for me. When I don't know what to eat, I also watch some short videos about it to learn. (male, 46 years)

#### 4.2.2 Self‐experiences

Respondents mentioned that changes in food choices were based on the lived experiences of some patients, and respondents developed these strategies by learning from the experiences of others, seeking advice or learning from their own experiences through a trial‐and‐error process. This strategy is evident in people who have had IBD for a long time.

Usually when I drink milk by myself, I have to have diarrhea after drinking it, then I know I am intolerant to this milk or what. (male, 37 years)

I personally experienced, like that more lubricating intestinal stuff, such as melon, melon, banana, eat will diarrhea. (male, 41 years)

#### 4.2.3 Traditional Chinese food cultures

Respondents attributed other explanations for dietary decisions to traditional Chinese food beliefs. Some participants used the terms ‘healthy diet’ and ‘clean diet’, and although these terms are not defined, assumptions can be made about the meaning of these terms. This tradition centres on the concept of yin and yang, which maintains a balance between the body and the environment. Foods are categorised as ‘hot’, ‘cold′ and ‘neutral’ according to their effect on the body (rather than their body temperature), and ‘fawu’ is ‘hot’ and should be avoided to maintain balance throughout the intestinal tract. In general terms, most participants believed that consuming foods classified as ‘fawu’ in Chinese medicine would worsen their bowel habits as patients, whereas eating lighter foods (‘qingdan’) would help, which may involve avoiding certain foods and cooking methods.

A patient said wolfberry plus infused water is a little better, I also listen to him, I hope to try to food supplement or food supplement. (male, 33 years)

The slimy vegetables are good for the intestines, so eat more of these vegetables. (female, 27 years)

### 4.3 ‘As I am allergic to eggs, I eat fish instead to get my protein’: Balancing expectations amidst limitations (Act)

#### 4.3.1 Positive adjustment: Repeated trials to find tolerable foods

Patient adherence with a nutrition plan entails frequent assessments, follow‐ups and attention to nuanced tastes and preferences. Respondents followed the recommendations of dietary guidelines and managed their symptoms by keeping a dietary diary or repeating trials to find tolerable food groups.

I keep a daily diet diary and slowly try new foods to see if I can tolerate them. I prefer to eat a variety of foods from the list of foods I want. (female, 44 years)

Occasionally when I eat noodles I add a little soy sauce, and the next day there is no reaction I know I am allowed to eat soy sauce. (female, 27 years)

Dietary decisions after illness have shifted to focus on meeting nutritional needs, with greater attention to ingredient mix and nutritional balance, and patients reported a pattern of replacing frozen or canned foods with fresh options in daily decision‐making.

There was only strawberry sauce and noodles at home, and I wanted to have a balanced nutrition, so I developed a new dish ‐ strawberry sauce with noodles, and unexpectedly it tasted good. (male, 29 years)

#### 4.3.2 Self‐limitation: Limit or avoid some foods

Patients reduced or eliminated red meat or processed meats after diagnosis because of the perceived relationship between these foods and health. However, some also cut back on nutritious foods such as vegetables and fruits.

I decided I should not eat too much food that is ‘toxic’ in nature, things like goose, duck, pigeons and chicken. Usually, I eat yams, carrots, cabbage, winter squash and pumpkin and that's it, nothing fancy. (male, 35 years)

#### 4.3.3 Static diets: No changes postdiagnosis

Few respondents reported that they had not made any dietary changes.

I had a healthy diet before I got sick, I don't like sweets and hot pot and all that, so now I basically have no change in food after I got sick. (male, 36 years)

### 4.4 ‘I feel like a blind man crossing a river’: Positive and negative perception (psychological experience)

#### 4.4.1 Keeping hope and enjoying

Some respondents reported that the ability to add more combinations and choices to their diet was a source of joy and hope for them as they became more aware of food.

I would be pleasantly surprised by the increasing amount of food I could eat as time went on and I believed I was in control of my food. (female, 28 years)

I received less information before, and since I joined various patient clubs in 2018 and got a lot of knowledge about diet management, I started to get more and more interested in food. (male, 29 years)

#### 4.4.2 Uncertainty

Despite the strong interest in IBD diet, reliable scientific information and daily dietary information to meet the needs of patients are relatively lacking. Patients do not have access to evidence‐based advice, and respondents report considerable uncertainty in daily decision‐making.

If you could not find your safe taste in food, you are, in fact, unsettled. I feel like a blind man crossing a river. (female, 44 years)

I felt uncertain about food, with one person telling me I could eat it and another telling me I couldn't, and there were no hard or fast rules to easily know what foods to eat and what to avoid. (female, 49 years)

## 5 DISCUSSION

This study led by a conceptual model of three decision‐making processes of naturalistic decision‐making showed that daily dietary decision‐making in adults with IBD includes monitoring, interpretating and acting phases. The monitoring phase provides insight into how patients use information about their gastrointestinal symptoms and nutritional status and food cues to make dietary decisions. The interpreting phase provides the patient's perspective on food choices and sensitivity to the source of knowledge. The acting phase provides prioritised behavioural events that patients make after evaluating the food. We also observed patients' complex and divergent psychosocial experiences during the monitoring, interpreting and acting phases of decision‐making. This study provides an important perspective on the food decision‐making process and psychological experiences of patients with IBD in healthcare research. To our knowledge, this study is the first to identify clear processes and perspectives from the patient's point of view when coping with diet‐related decisions from monitoring to interpretation to action.

Dietary decisions in patients with IBD occur in a dynamically evolving real‐world environment. Health and flavour are two important factors that we consider when selecting food. In general, when we want to eat healthier, we will either increase our health consciousness or decrease our desire for delicious food. However, there appeared to be less consistency amongst patients with IBD, whose perceptions of the severity of gut inflammation and food cues influenced dietary decisions through reprioritisation. Some patients reported that they were willing to give up values such as food enjoyment to ensure bowel stability because they perceived their condition to be severe and more disruptive to their lives. Meanwhile, patients with IBD believe that diet is important in managing bowel symptoms and often seek additional guidance on this matter from outside sources.^11^ However, some of the patients in this study reported that dietary advice provided by healthcare professionals was often broad, generalised and even contradictory. A previous cross‐sectional study^12^ on the quality of care for patients with IBD reported that satisfaction was the lowest for ‘dietary advice from a dietitian’. Furthermore, although gastroenterologists are experts in the function of the gastrointestinal tract, most of them are not experts in nutritional science. Most physicians receive minimal training during medical school in nutrition, and the minority with the required dietary expertise frequently lack the chance to engage with patients on a longitudinal basis to create a long‐term change. This factor may lead to increased confusion and bewilderment amongst patients about complex dietary choices. According to previous studies,^13^, ^14^ many patients with IBD employ an elimination diet to avoid disease exacerbation. Such behaviour affects their social life by limiting the occasions when meals are eaten outside the home or in eating different meals to other household members. Consistent with the findings of this study, avoidance or dietary restriction will be prioritised when faced with uncertain dietary decisions. Such a high percentage of patients with IBD employing dietary restrictions during remission has not been confirmed by the recommendations formulated by various gastroenterology organisations. In their recommendations for patients with CD, the ESPEN guidelines^15^ advise a diversified and well‐balanced diet, without addressing a detailed composition. Hence, early specific and in‐depth dietary information would increase patient knowledge and could prevent the adoption of unjustified exclusion diets.

Nutritional perceptions and beliefs are fundamental factors influencing food choices.^16^ This is even more important for Chinese patients with IBD. The dynamics of the disease and the complexity of diet indicate that healthy dietary recommendations for people with IBD are neither absolute nor universal. In this study, participants' food choices appeared to be guided by their understanding of modern Western and Chinese medicine nutritional concepts. The findings suggest that the stronger their nutritional beliefs, the more confident they were in describing and justifying their food choices. It was also clear that when participants fully grasped a nutritional belief, they overcame environmental barriers to practice that belief. This is consistent with other studies showing that nutritional beliefs are related to dietary choices.^16^, ^17^ Some studies have found that patients are also likely to follow an ‘unguided’ diet (meaning no guidance from a physician or dietitian), where daily dietary choices are based on patients’ perceptions of foods or on the experiences of others.^18^, ^19^ Knowledge from professionals may not be comprehensive and detailed enough, and patients will form the basis for their own dietary decisions after receiving expertise and combining it with their own dietary experience.^20^ One participant described their decision‐making around dietary choices as ‘changing the foods I naturally eat, without even thinking about it, and then becoming more aware.’ This process can also be described as perceptual eating.^17^ When participants discover which foods help reduce their symptoms, they intuit which foods cause them to experience symptoms and which do not based on their knowledge or sense of knowledge. Perceptual eating can include the decision‐making process of weighing the emotional and physiological effects of certain foods before choosing whether or not to eat them. Over time, with the knowledge and experience gained through the experimental process, they re‐established their interaction with food and believed that they could conduct their own research and know what was best for them. Reaching this state of acceptance is a long process because of the lack of professional guidance. Personal dietary philosophies have rarely been mentioned in previous studies, but are important determinants of food choices.^21^ In this study, the dietary philosophy that participants eat to be healthy was more prominent. The dietary philosophy of individuals with IBD appears to influence food choice in an indirect way. It seems to be an inherent attitude that sheds light on many aspects of food choice. For example, in the current study, participants who considered eating as a life pleasure before becoming ill shared that they spent more time and money on food, enjoyed talking about food, and rarely chose food because of health concerns. In contrast, after getting sick, more consideration was given to the health of the food rather than its flavour. Thus, food philosophy seems to be closely related to individuals' motivations towards food.^22^ Given that different types of dietary philosophies exist for different disease states and types of patients, there is a need to investigate patients' dietary philosophies and then provide tailored nutritional education messages or interventions that are more effective. It is unrealistic to expect healthcare professionals to have all the answers or to develop comprehensive and individualised interventions for each patient. Therefore, the first important step would be to promote a supportive space where patients' voices are heard and difficulties are normalised in the context of suffering from challenging dietary decisions.

The majority of Chinese patients with IBD interviewed had adopted an avoidance or restrictive approach to their diets, focussing on avoiding single foods rather than improving their overall diets, because of the influence of the causality of their bowel symptoms and traditional Chinese medicine (TCM) beliefs, which are prominent in this population. Beliefs associated with TCM and its hot/cold dichotomy influence dietary decisions, with patients consuming or avoiding foods based on their properties. The gut maintains balance in different ways, including food behaviour (i.e., eating or avoiding certain foods). In Chinese culture, food therapy is a method of adjusting diet and using food to promote health based on TCM theories. TCM nutrition is an ancient but burgeoning discipline, and it primarily aims to use food as a means to achieve balance and harmony within the body. Compared with modern nutrition, TCM nutrition has unique beneficial concepts, such as holism, diet suggestions based on syndrome differentiation, the idea that the spleen and stomach are the ‘root’ of post‐heaven and the homology of medicine and food. To date, evaluating whether TCM nutrition could play a major role in the treatment of various diseases is difficult.^23^ TCM and Western nutritional science are two completely separate disciplines that provide guidance for healthy eating. Previous systematic reviews^21^ have shown that Chinese people encounter conflicting dietary advice information from TCM and modern Western nutritional sciences Inconsistency in dietary advice inevitably confuses Chinese people and may lead to distrust of some Western nutritional advice. However, these strong cultural beliefs created social pressure to adhere to cultural norms, including the consumption of light foods and the avoidance of ‘hairy’ foods in the postoperative period.^24^ Notably, ‘hairy items’ is actually a traditional Chinese medical science concept, and whether it worsens the gut remains uncertain. In addition, knowledge of how diet affects IBD may be influenced by lay sources. Although the socio‐cultural context is often discussed extensively in the context of behavioural change, its importance cannot be ignored.^25^ Finally, it is worth noting that there are also differences in health perceptions and practices across China, which influence the way patients eat. In our study, the most significant differences were found between people from northern and southern China, which is consistent with the wide variation in Chinese cooking.^26^ In southern China, patients usually drank more soup and avoided raw vegetables, but this was not always the case for patients from northern China. The limitations of patients' interpretation of dietary decisions based on TCM nutrition primarily include the limited scope of application, lack of evidence‐based research and the need for the cooperation of TCM clinicians. In contemporary China, the government should establish a system in which TCM nutrition and modern nutrition coexist, and TCM nutritionists should be provided with higher levels of specialist training. In addition, TCM nutrition should integrate the research methods of modern nutrition in IBD, adjust for the target population and formulate age‐specific nutritional principles to reflect the advantages and characteristics of TCM nutrition.

This study was conducted in a relatively broad population of patients with IBD. In addition, this study recruited patients consecutively by disease activity as measured by clinical scores and included a heterogeneous disease course and dietary culture. However, this study also has some limitations. Firstly, the sample population was Chinese adults with IBD, which may affect the results because patients of different races and countries may have different symptoms and responses to food and have different experiences in food decision‐making. Other limitations include the length of the interviews. Patients with IBD may become fatigued and less candid about long interviews. Secondly, all respondents were adult patients aged 18–60 years. These patients may experience and feel differently during IBD dietary decision‐making compared with those of other ages. Moreover, the duration of the disease varies widely amongst patients, and differences in the duration of the disease may affect dietary decisions. Thirdly, the interview process may be subject to recall bias. The interviews involved dietary changes and decision‐making processes before and after diagnosis. The causal relationship of food triggers is difficult to determine, and it can change over time. Patients are recruited during remission or activity, and the response process and psychological experience of dietary decisions may also be influenced by the course of the illness before the interview.

## 6 CONCLUSION

To our knowledge, this study is the first qualitative study on the dietary decision‐making process of patients with IBD. This study shares the monitoring, interpreting and acting phases of the dietary decision‐making process of patients with IBD. The result of this study can facilitate healthcare providers and policy makers to obtain a comprehensive understanding of food‐related challenges faced by patients with IBD and to find feasible ways to increase support for and with patients. Therefore, improving the dietary health of people with IBD requires not only greater health education, but also the incorporation of healthy dietary goals into personal and social decision‐making priorities. Furthermore, incorporating healthy dietary considerations into everyday issues is the crux of the matter.

## AUTHOR CONTRIBUTIONS

All authors took part in the conception, design and analysis of data. In addition, data collection was carried out by Yin Ting‐Ting and Tu Wen‐Jing. Drafting of the manuscript was performed by Yin Ting‐Ting and Li Yi‐Ting. Critical revisions for important intellectual content were carried out by Tu Wen‐Jing and Xu Wen‐Jing. Xu Gui‐Hua and Tu Wen‐Jing contributed to the study design and setup. Tu Wen‐Jing and Yin Ting‐Ting coded and analysed the data. All authors were involved in the generation of final themes and codes. All authors have agreed on the final version of the manuscript.

## CONFLICT OF INTEREST STATEMENT

The authors declare no conflict of interest.

## ETHICS STATEMENT

The study was conducted in accordance with the Declaration of Helsinki and approved by the Ethics Committee of Nanjing Hospital of Traditional Chinese Medicine (KY2022029, approval date: 25 February 2022). Registration was completed at the China Clinical Trials Registry (ChiCTR2200064943, registration date: 24 October 2022). Informed consent was obtained from all subjects involved in the study.

## Supporting information

Supporting information.

Supporting information.

## Data Availability

Data are not freely available.
