# Supplementary material for: ‘Eating is like experiencing a gamble’: A qualitative study exploring the dietary decision‐making process in adults with inflammatory bowel disease
Source: Health Expect. 2023 Sep 20;27(1):e13873. doi: 10.1111/hex.13873 (PMC10726150; doi:10.1111/hex.13873)
Supplement: Supplementary file 1 — Supporting information. [file HEX-27-e13873-s002.docx]

**Interview topic guide**

**Introduction**

Interviewer introduces herself, and the aims of the study. Ground rules:

- Participant is free to state at any time if they feel uncomfortable with questions or want to stop the interview.
- The interview will be audio recorded and the interviewer will make brief notes. Both will be anonymised after the interview.
- There are no right or wrong answers and all responses are valid.

**General questions**

- First, I want to learn more about your current basic information. Please tell me your age, gender,weight, height, ethnicity, marital status, educational qualification, and employment status.
- Second, I want to learn more about your current characteristics of the disease. Please tell me your subtypes of IBD, past and current surgical interventions, medications, and duration of the disease, medicaments, and disease activity at the time of examination were recorded.
- Third, I want to learn more about your current nutritional status.Please tell me your current weight, height, body mass index (BMI), presence of food allergies or intolerances, and use of dietary supplements.

**Determinants**

- Have you changed your diet since your diagnosis?
- If it has changed, what and how has changed?
- How do you make decisions about food choices?
- What were your feelings or emotions during the dietary decision-making process?
- What is the basis of the decision?
- What factors influence your decision making?

**Wrap up**

- Is there anything else you’d like to mention that we haven’t covered?
- Close; thank the participant; and provide debriefing sheet.
