# Supplementary material for: ‘Eating is like experiencing a gamble’: A qualitative study exploring the dietary decision‐making process in adults with inflammatory bowel disease
Source: Health Expect. 2023 Sep 20;27(1):e13873. doi: 10.1111/hex.13873 (PMC10726150; doi:10.1111/hex.13873)
Supplement: Supplementary file 2 — Supporting information. [file HEX-27-e13873-s001.docx]

Consolidated criteria for reporting qualitative studies (COREQ): 32-item checklist

| **No** | **Item** | **Guide questions/description** |
| --- | --- | --- |
| **Domain 1: Research team and reflexivity** | | |
| Personal Characteristics | | |
| 1. | Interviewer/facilitator | TY |
| 2. | Credentials | TY(MSc), WT(PhD), YL (MSc), WX(MSc),GX(PhD) |
| 3. | Occupation | TY(Student),WT(Inflammatory bowel disease and Research Specialist), LW (Student), WX(Student), GX (Professor of Nursing) |
| 4. | Gender | female |
| 5. | Experience and training | The team members have all received rigorous study and training in qualitative research, and TY has some experience with qualitative interviews. |
| Relationship with participants | | |
| 6. | Relationship established | Yes. |
| 7. | Participant knowledge of the interviewer | Few participants were known to the YT. |
| 8. | Interviewer characteristics | All interviewers are public nutrition and inflammatory bowel disease researchers and are particularly interested in qualitative methods. |
| **Domain 2: Study design** | | |
| Theoretical framework | | |
| 9. | Methodological orientation and Theory | descriptive phenomenology and thematic analysis |
| Participant selection | | |
| 10. | Sampling | Purposive and convenience sampling |
| 11. | Method of approach | We conducted five face-to-face field interviews.15 interviews were conducted online via Wechat or over the phone because of the COVID-19 pandemic. |
| 12. | Sample size | 20 |
| 13. | Non-participation | Zero.We purposively selected participants for interview. And all participants agreed to give an interview after we approached them. |
| Setting | | |
| 14. | Setting of data collection | Word Office |
| 15. | Presence of non-participants | No |
| 16. | Description of sample | See Table 1 |
| Data collection | | |
| 17. | Interview guide | An interview guide was drafted, piloted，see supporting information appendix |
| 18. | Repeat interviews | None |
| 19. | Audio/visual recording | All interviews were audio-recorder. |
| 20. | Field notes | Yes |
| 21. | Duration | 39–152 minutes |
| 22. | Data saturation | Yes |
| 23. | Transcripts returned | Yes |
| **Domain 3: Analysis and findings** | | |
| Data analysis | | |
| 24. | Number of data coders | No |
| 25. | Description of the coding tree | See Table 2 |
| 26. | Derivation of themes | Theme were derived from the data based on Colaizzi seven-step analysis |
| 27. | Software | No |
| 28. | Participant checking | Yes |
| Reporting | | |
| 29. | Quotations presented | Yes |
| 30. | Data and findings consistent | Several relevant quotations used to illustrate findings. |
| 31. | Clarity of major themes | Yes |
| 32. | Clarity of minor themes | Yes |
